# A diarylamine derived from anthranilic acid inhibits ZIKV replication

**DOI:** 10.1038/s41598-019-54169-z

**Published:** 2019-11-27

**Authors:** Suely Silva, Jacqueline Farinha Shimizu, Débora Moraes de Oliveira, Leticia Ribeiro de Assis, Cintia Bittar, Melina Mottin, Bruna Katiele de Paula Sousa, Nathalya Cristina de Moraes Roso Mesquita, Luis Octávio Regasini, Paula Rahal, Glaucius Oliva, Alexander Luke Perryman, Sean Ekins, Carolina Horta Andrade, Luiz Ricardo Goulart, Robinson Sabino-Silva, Andres Merits, Mark Harris, Ana Carolina Gomes Jardim

**Affiliations:** 1 Laboratory of Virology, Institute of Biomedical Science, ICBIM, Federal University of Uberlandia, Uberlândia, MG Brazil; 2 São Paulo State University, IBILCE, S. José do Rio Preto, SP Brazil; 3 Laboratory of Antibiotics and Chemotherapeutics, São Paulo State University, IBILCE, S. José do Rio Preto, SP Brazil; 4 LabMol - Laboratory of Molecular Modeling and Drug Design, Faculdade de Farmácia, Universidade Federal de Goias, Goiânia, GO 74605- 170 Brazil; 5 Institute of Physics of São Carlos, University of São Paulo, São Carlos, Brazil; 6 Department of Pharmacology, Physiology and Neuroscience, Rutgers University–New Jersey Medical School, Newark, NJ 07103 United States; 7 Present Address: Repare Therapeutics, 7210 Rue Frederick-Banting, Suite 100, Montreal, QC H4S 2A1 Canada; 8 Collaborations Pharmaceuticals, Inc., 840 Main Campus Drive, Lab 3510, Raleigh, NC 27606 United States; 9 Laboratory of Nanobiotechnology, Federal University of Uberlandia, Uberlândia, MG Brasil; 10 Integrative Physiology and Salivary Nanobiotechnology Group, Federal University of Uberlandia, Uberlândia, MG Brasil; 11 Institute of Technology, University of Tartu, Nooruse 1, 50411 Tartu, Estonia; 12 School of Molecular and Cellular Biology, Faculty of Biological Sciences and Astbury Centre for Structural Molecular Biology, University of Leeds, Leeds, LS2 9JT United Kingdom

**Keywords:** Drug screening, Virology

## Abstract

Zika virus (ZIKV) is a mosquito-transmitted Flavivirus, originally identified in Uganda in 1947 and recently associated with a large outbreak in South America. Despite extensive efforts there are currently no approved antiviral compounds for treatment of ZIKV infection. Here we describe the antiviral activity of diarylamines derived from anthranilic acid (FAMs) against ZIKV. A synthetic FAM (E3) demonstrated anti-ZIKV potential by reducing viral replication up to 86%. We analyzed the possible mechanisms of action of FAM E3 by evaluating the intercalation of this compound into the viral dsRNA and its interaction with the RNA polymerase of bacteriophage SP6. However, FAM E3 did not act by these mechanisms. *In silico* results predicted that FAM E3 might bind to the ZIKV NS3 helicase suggesting that this protein could be one possible target of this compound. To test this, the thermal stability and the ATPase activity of the ZIKV NS3 helicase domain (NS3^Hel^) were investigated *in vitro* and we demonstrated that FAM E3 could indeed bind to and stabilize NS3^Hel^.

## Introduction

Zika virus (ZIKV) is a mosquito - transmitted virus first isolated in 1947 from a *Rhesus* monkey in the Zika forest, Uganda^1^. ZIKV remained endemic to the African and Asian regions until 2007, since then the virus has spread to other continents^2–6^. Notably, in 2015, the ZIKV outbreak had a worldwide impact and was considered a serious public health problem due to the large number of people infected and the development of neurological disorders in neonates (microcephaly) and adults (Guillain Barre syndrome)^7^.

Similar to other arboviruses such as Dengue virus (DENV), Yellow Fever virus (YFV) and Chikungunya virus (CHIKV), ZIKV is mainly transmitted by *Aedes spp*. of mosquitoes^8–10^. Nevertheless, other sources of infection acquisition have been reported, including blood transfusion^9^, sexual^11,12^, perinatal and transplacental transmissions^5,13^. Recently, it has been suggested that ZIKV may also have a sylvatic transmission cycle which could increase the frequency of human reinfection^14^.

ZIKV belongs to the *Flaviviridae* family and genus *Flavivirus*^15^. As other members of the genus, the viral genome is a positive single-stranded RNA with one open reading frame (ORF), translated in a polyprotein that is cleaved by host and viral proteases into 10 proteins. The polyprotein yields seven nonstructural proteins involved in the viral replication process (NS1, NS2A, NS2B, NS3, NS4A, NS4B and NS5), and three structural proteins (capsid (C), pre-membrane (prM) and the envelope (E) proteins), which comprise the viral particles^16–18^.

There are currently no approved antiviral compounds targeting ZIKV infection. The treatment of infected individuals is palliative and consists of fluid intake and rest. Therefore, there is an urgent need for research to develop effective antivirals. In this context, the therapeutic properties of natural compounds have been historically described for the treatment of several viral diseases, such as hepatitis C virus (HCV)^19,20^, human immunodeficiency virus (HIV-1)^21^, CHIKV^22^, DENV and West Nile virus (WNV)^23^. Natural products present advantages such as high chemical diversity, low cost of production and efficient metabolism^24,25^. However, compounds isolated from natural sources are not patentable and the isolation process is time consuming^24,26^. An attractive alternative is to use the structure of the natural products as scaffolds for the synthesis of new molecules that can enhance the bioactivity and are more amenable to large scale manufacture^26–28^.

Natural and synthetic acids have attracted attention due to their potent antiviral properties. This is exemplified by glycyrrhizic acid which prevents the release of HCV infectious particles^29^ and inhibits hepatitis A virus (HAV) penetration into the host cell^30^, and 3,5-dicaffeoylquinic acid, 1-methoxyoxalyl-3,5-dicaffeoylquinic acid, and L-chicoric acid that were described to prevent HIV-1 viral replication^31^. Similarly, nordihydroguaiaretic acid (NDGA) and its derivative tetra-o-methyl nordihydroguaiaretic acid were demonstrated to block DENV, HCV^32^, WNV and ZIKV^33^ replication.

Here we evaluated the antiviral activity of synthetic diarylamines derived from anthranilic acid (FAMs) on ZIKV infection *in vitro* and *in silico*. Our data showed that FAM E3 significantly inhibited the ZIKV genome replication.

## Results

### ZIKV infection can be inhibited by synthetic FAMs

**Figure 1:**
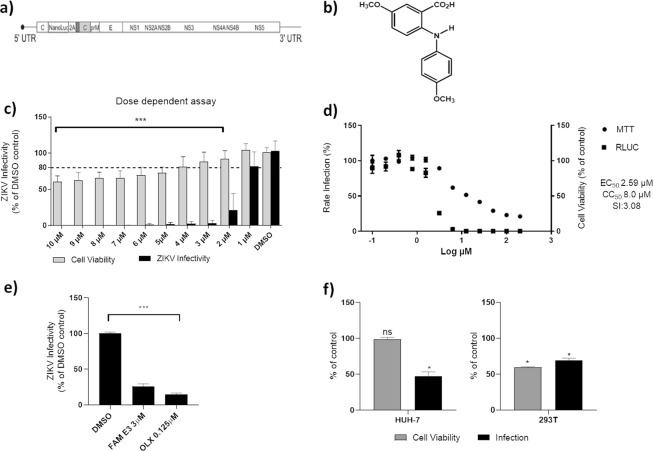
Inhibitory activity of FAM E3 on ZIKV replication. Schematic representation of ZIKV-Nanoluc that continuously expresses the Nanoluciferase reporter (**a**). Chemical structure of FAM E3 (**b**). Dose response assay: ZIKV-Nanoluc infected cells (MOI 0.1) were treated with FAM E3 at concentrations ranging from 1 to 10 µM and virus replication efficiency was evaluated 72 h.p.i. Simultaneously, Vero cells were equally treated with FAM E3 and cells viability was measured 72 h later (**c**). Effective and cytotoxic concentration of 50%: Vero cells were treated with increasing concentrations of FAM E3 ranging from 0.10 to 200 µM. ZIKV replication was measured by luciferase assay (indicated by ■) and cellular viability measured using an MTT assay (indicated by ●) (**d**). Vero cells were infected with ZIKV^BR^ and treated with FAM E3 at 3 µM and virus replication was accessed 72 h.p.i. The intracellular virus was titrated by analysing focus- forming units per milliliters (Ffu/mL), DMSO and OLX were used as negative and positive controls, respectively (**e**). Huh-7 or 293 T cell lines were infected with ZIKV-Nanoluc and treated with FAM E3 (3 µM) or DMSO (0.1%) for 72 h (**f**). Mean values of three independent experiments each measured in quadruplicate including the standard deviation are shown. P < 0.05 was considered significant compared to DMSO control.

A panel of FAMs synthesized based on natural scaffolds was screened using a recombinant ZIKV that expresses the Nanoluciferase reporter (ZIKV-Nanoluc) (Fig. 1A). This recombinant virus was shown previously to exhibit a similar replication rate to wild type virus^34^. To assess cytotoxicity, Vero cells were treated with differing concentrations of each FAM (0.4, 2, 10 and 50 μM) and cell viability was measured 72 h post-treatment. Then, Vero cells were infected with ZIKV-Nanoluc at a MOI of 0.1 in the presence or absence of each compound at specific concentrations and Nanoluciferase activity levels, proportional to viral replication, were assessed 72 h post infection (h.p.i).

From all compounds evaluated, FAM E3 (Fig. 1B) showed the highest inhibition rate (Table S1). We therefore performed a dose response assay to determine effective concentration 50% (EC_50_) and cytotoxicity 50% (CC_50_) values for FAM E3. ZIKV-Nanoluc infected Vero cells were therefore treated with FAM E3 at concentrations ranging from 1 to 10 µM and virus replication efficiency was evaluated 72 h.p.i. In parallel cell viability was measured by MTT assay. Our data showed that FAM E3 was able to inhibit >99% of virus replication, while the minimum cell viability remained above 60% **(**Fig. 1C). Using a wider range of FAM E3 concentrations, it was determined that the compound has an EC_50_ of 2.59 µM, CC_50_ of 8.0 µM and SI of ~3 (Fig. 1D). For further analysis, cells were treated with FAM E3 at 3 µM, which inhibited approximately 96% of ZIKV infectivity with cell viability >88% (Fig. 1C). To confirm that the activitiy of FAM E3 against ZIKV was not specific to the laboratory isolate, it was also tested against a primary clinical isolate of ZIKV (provided by the Evandro Chagas Institute in Belém, Pará^35^) (ZIKV^BR^). For this, Vero cells were infected with ZIKV^BR^ at MOI = 0.1 and treated with FAM E3 3 µM or controls for 72 h. Then, intracellular virus was titrated by analysing focus-forming units per milliliters (Ffu/mL). The results corroborated to the data from ZIKV-Nanoluc (Fig. 1E).

The antiviral effect of FAM E3 was also investigated in the ZIKV human permissive cell lines Huh-7 and 293 T. Infected cells were treated with 3 µM of FAM E3 and both cell viability and ZIKV infectivity were evaluated. The results showed that FAM E3 was able to significantly decrease ZIKV replication levels in both cell types (Fig. 1F). However, 293 T cells appeared to be acutely sensitive to the cytotoxic effect of FAM E3.

### FAM E3 inhibits the post-entry stage of ZIKV replication

**Figure 2:**
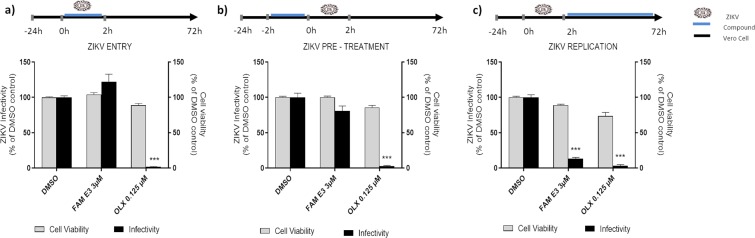
Effects of FAM E3 on the different stages of the ZIKV replicative cycle. Vero cells were infected with ZIKV-Nanoluc at a MOI = 0.5 and simultaneously treated with FAM E3 for 2 h; cells were washed to remove the virus and replaced with fresh media. ZIKV replication was measured by Nanoluc activity at 72 h.p.i (**a**). Vero cells were treated with FAM E3 for 2 h. Then, cells were extensively washed and infected with ZIKV-Nanoluc at a MOI = 0.5 for 2 h. The inoculum was removed and the cells were washed and replaced with fresh media. ZIKV replication was measured by Nanoluc activity at 72 h.p.i. (**b**). Vero cells were infected with ZIKV-Nanoluc at a MOI = 0.5 for 2 h. The virus was removed, cells were washed and added of fresh media containing FAM E3. ZIKV replication was measured by Nanoluc activity at 72 h.p.i (**c**). For all assays, non-infected Vero cells were equally treated with FAM E3 and cell viability was measured 72 h later using MTT assay. DMSO was used as negative control and OLX as positive control for infectivity inhibition. Mean values of three independent experiments each measured in quadruplicate including the standard deviation are shown. P < 0.05 was considered significant.

To analyze the effects of FAM E3 on different stages of the ZIKV replicative cycle, time of addition experiments were performed. To evaluate the activity of compound on virus entry, FAM E3 and ZIKV-Nanoluc were simultaneously added to the cells for 2 h at 37 °C. Then, the inoculum was removed, cells were extensively washed with PBS, fresh media was added, and the cells were incubated for 72 h (Fig. 2A). In contrast to the control obatoclax (OLX) that is known to inhibit the entry of ZIKV^36^, the results showed that FAM E3 had no effect on ZIKV entry into the host cell (Fig. 2A).

We further investigated whether FAM E3 could elicit a protective effect. For this, cells were pretreated by incubation in medium containing FAM E3 for 2 h. prior to infection with ZIKV-Nanoluc for 72 h as shown in Fig. 2B, FAM E3 had no significant effect on ZIKV infection, suggesting that this compound is not acting by rendering the cells refractory to infection with ZIKV (Fig. 2B).

Finally, we analyzed the effect of FAM E3 on post-entry steps of ZIKV infection. For this, Vero cells were incubated with ZIKV-Nanoluc for 2 h, and then the inoculum was replaced by medium containing FAM E3. The data showed that FAM E3 decreased viral replication up to 86% whilst retaining cell viability above 90% (Fig. 2C). These data suggest that the antiviral activity of FAM E3 is related to its ability to inhibit a post-entry stage of the virus lifecycle, most likely viral RNA replication.

### Potential mechanisms of action of FAM E3

**Figure 3:**
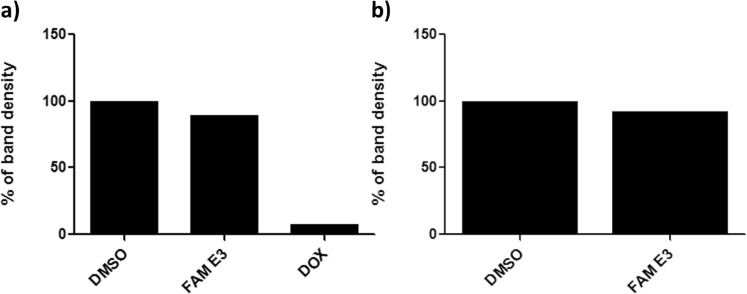
Analysis of FAM E3 intercalation into the viral dsRNA and its interaction with the activity of phage SP6 RNA polymerase. Fifteen nanomoles of dsRNA were incubated with the FAM E3 or intercalating controls (DMSO) or (DOX) for 45 minutes at room temperature. The reaction products were subjected to 1% agarose electrophoresis gel containing Ethidium Bromide followed by densitometry analysis (**a**). FAM E3 and 5 µg of purified pCCI-SP6-ZIKV amplicon was used for *in vitro* transcription using SP6 RNA polymerase at the presence or absence of FAM E3. Reaction products were analysed by agarose gel electrophoresis followed by densitometry analysis (**b**). Results of a representative of three independent reproducible experiments are shown.

To investigate possible mechanisms of action of FAM E3, we analyzed the ability of FAM E3 to intercalate into dsRNA, the replication intermediate of all positive-strand RNA viruses. Fifteen nanomoles of an *in vitro* synthesized dsRNA was incubated with FAM E3 or controls (DMSO or the well characterized intercalating agent doxorubicin (DOX)) and the obtained RNA/compound complexes were analyzed in 1% agarose gel. Densitometry analysis showed that FAM E3 did not intercalate with dsRNA (Fig. 3A).

As an assay for the RNA-dependent RNA polymerase activity of ZIKV NS5 was not available, we attempted to elucidate whether FAM E3 interacts with RNA synthesis carried out by the unrelated bacteriophage SP6 DNA-dependent RNA polymerase. For this, an *in vitro* transcription assay using SP6 RNA polymerase was performed in the presence or absence of FAM E3. Reaction products were analyzed using agarose gel electrophoresis and densitometry. As shown in Fig. 3B FAM E3 was unable to inhibit synthesis of ZIKV RNAs by SP6 RNA polymerase.

**Figure 4:**
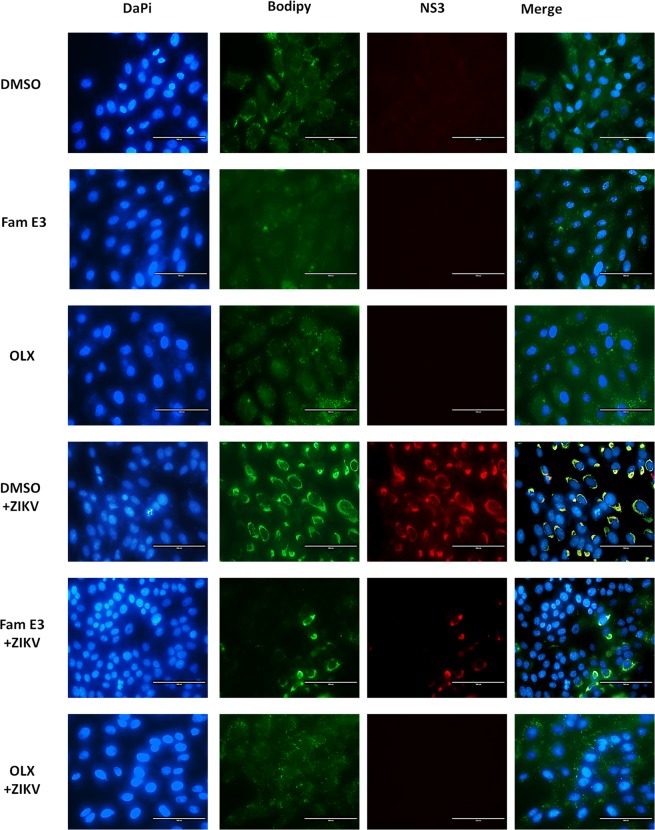
FAM E3 interference with the cell lipid metabolism of the host cells. Vero cells were infected with ZIKV at MOI = 0.1 and treated with FAM E3 3 µM or DMSO 0.1% or OLX controls for 72 h. Naïve Vero cells were treated with DMSO were used as non-infected cells control. After treatment, cells were fixed and nuclei, lipid droplets (LDs) and ZIKV NS3 were labeled using DAPI (blue), BODIPY 493/503 (green) and ZIKV anti-NS3 antibody (red), respectively. Scale bar 100 nm.

To test whether FAM E3 interfered with the cell lipid metabolism of the host cells. Vero cells infected with ZIKV-Nanoluc and treated with FAM E3, DMSO or OLX were fixed and stained with DAPI (to detect nuclear DNA), Bodipy to detect lipid droplets and an anti-NS3 antibody. As expected ZIKV infection increased lipid droplet accumulation and this was reduced by FAM E3 treatment, However, FAM E3 did not significantly reduce lipid droplet accumulation in non-infected Vero cells. Based on this result, the decrease in lipid droplets in infected Vero cells treated with FAM E3 is likely a consequence of the inhibition of virus replication, suggesting other mechanism of action for FAM E3 (Fig. 4).

### FAM E3 is able to bind to and stabilize the ZIKV NS3^Hel^ protein

**Figure 5:**
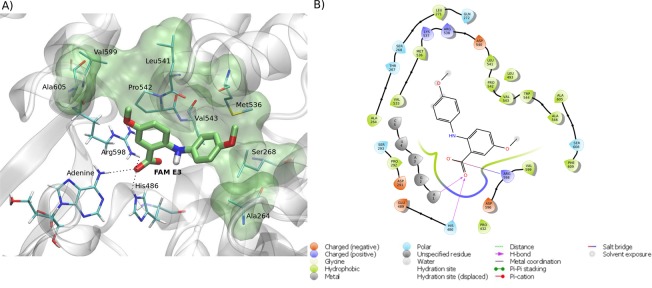
Predicted intermolecular interactions between FAM E3 and the RNA binding site of ZIKV NS3^Hel^. 3D structure of the RNA binding site of ZIKV NS3^Hel^ docked with FAM E3, highlighting the main interactions between FAM E3 and amino acid residues, through hydrogen bonds (dotted black lines) and hydrophobic interactions (transparent green surface) (**a**). 2D representation of the protein-ligand interactions (**b**).

**Figure 6:**
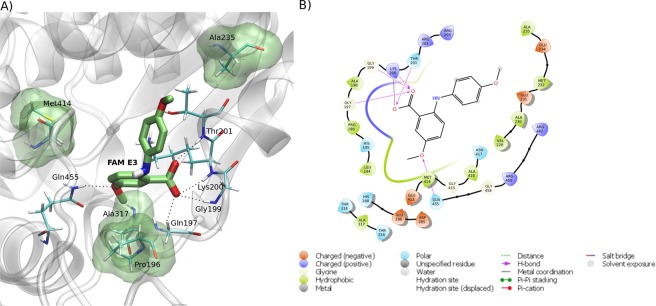
Predicted intermolecular interactions between FAM E3 and the ATP binding site of ZIKV NS3^Hel^. 3D structure of the ATP binding site of ZIKV NS3^Hel^ docked with FAM E3, highlighting the main interactions between FAM E3 and amino acid residues, through hydrogen bonds (dotted black lines) and hydrophobic interactions (transparent green surface) (**a**). 2D representation of the protein-ligand interactions (**b**).

Molecular docking calculations were performed in order to investigate the possible binding mode and the interactions between FAM E3 and ZIKV proteins. The proteins NS2B-NS3 protease, NS3 helicase, NS5 methyltransferase and NS5 polymerase, capsid and envelope were selected due to the availability of their experimentally obtained 3D structures in the protein data bank (PDB). The two best docking scores were obtained for NS3 helicase (NS3^Hel^) (−8.7 and −7.8 Kcal·mol^-1^, for RNA and ATP binding sites, respectively) (Figs. 5 and 6). As shown in Fig. 5, FAM E3 is predicted to bind into the NS3^Hel^ RNA binding pocket: the carboxylic acid moiety of FAM E3 participating in hydrogen bonding interactions with the amino acid residues Arg598, His486 and adenine (A1) (Fig. 5A,B). Moreover, the aromatic rings and hydrophobic groups of FAM E3 were predicted to make hydrophobic packing interactions with residues Ala264, Ser268, Met536, Leu541, Pro542, Val543, Val599 and Ala605 (Fig. 5A,B).

FAM E3 was also predicted to bind into the NS3^Hel^ ATP binding pocket (Fig. 6), in this case the carboxylic acid moiety of FAM E3 is predicted to form hydrogen bonding interactions with the amino acid residues Gln197, Gly199, Lys200 and Thr201. The ether group of FAM E3 can make a hydrogen bond with Gln455 (Fig. 6A,B). It is noteworthy that a similar interaction is observed in the crystal structure of NS3^Hel^, between the Gln455 residue and ATP^37^. Moreover, FAM E3 made hydrophobic interactions with the residues Pro196, Ala235, Ala317 and Met414 (Fig. 6A,B).

**Figure 7:**
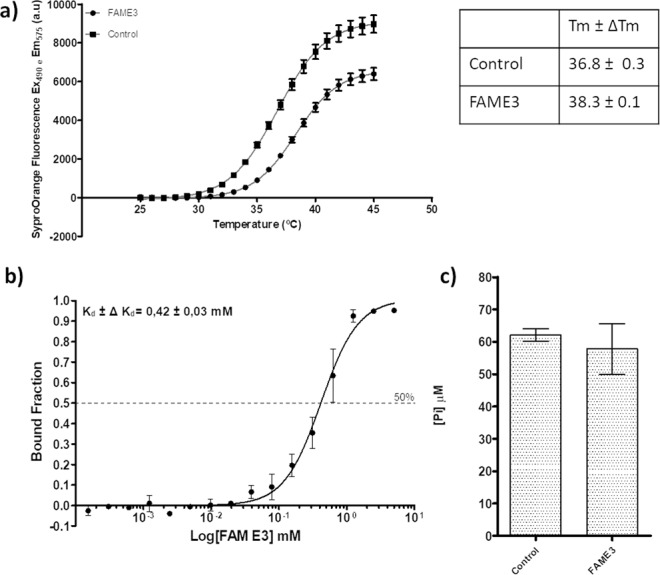
FAM E3 activity on NS3^Hel^. Thermal denaturation curves of NS3^Hel^ and Boltzmann fitting in the presence of DMSO (Control) or FAM E3. Tm of NS3^Hel^ obtained after Boltzmann fitting is represented on the table (**a**). MicroScale Thermophoretic analysis of the interaction between FAM E3 and NS3^Hel^. Data from four experiments were normalized to the fraction of bound ligand and averaged. Kd constant was obtained after a sigmoidal shape fitting with the Hill function (black continuous line) (**b**). NTPase activity of ZIKV NS3^Hel^ in the presence or absence of FAM E3 and using ATP as substrate (**c**).

In order to experimentally validate the results obtained by docking calculations, a thermal stability assay of the ZIKV NS3^Hel^ domain was performed by Differential Scanning Fluorimetry (DSF). The thermal denaturation curves of NS3^Hel^ in the presence of FAM E3 or non-treated control showed that FAM E3 was able to increase the NS3^Hel^ melting temperature (Tm). This data suggests that FAM E3 could bind to and stabilize the NS3^Hel^ protein (Fig. 7A). Additionally, a Micro-Scale Thermophoresis assay was carry out to evaluate the affinity of FAM E3 by NS3^Hel^. As observed in Fig. 7B, a sigmoidal slope was obtained, showing that FAM E3 could bind to NS3^Hel^. A fitting with the Hill function, a K_d_ of (0,42 ± 0,03) mM was obtained into the *in vitro* assay, showing a low affinity of the compound to the protein, in absence of ATP on NTP binding site. Finally, the effect of FAM E3 on the NTPase activity of NS3^Hel^ was also investigated by performing an NTPase activity assay using ATP as substrate and analyzing the amount of phosphate released by the protein in the reaction. FAM E3 did not significantly decrease the amount of phosphate released, compared to the non-treated control (Fig. 7C).

## Discussion

In this study we evaluated the ability of synthetic diarylamines derived from anthranilic acid (FAMs), designed based on their natural scaffolds, to inhibit ZIKV infection. From this screen we selected FAM E3 for further analysis as it demonstrated the highest level of inhibition against ZIKV. FAM E3 is an intermediary molecule obtained during the production of synthetic acridones. The chemical structure of the FAMs is based on two aromatic rings and one hydrogen atom linked to an amine group^38^. Our results demonstrated that FAM E3 was able to inhibit ZIKV by blocking viral RNA replication, but it had no effect on ZIKV cell entry.

The observed inhibition of ZIKV RNA replication may result from different biological effects of FAM E3, including effects on viral RNA polymerase activity, interference with replicase complex formation, and suppression of interaction of viral replicase proteins with host components. Previous studies have shown that acid derivative-containing compounds have interfered in the replicative cycle of different virus families. Zanello and coworkers showed that N-sulfonyl anthranilic acid derivatives inhibited the replication of DENV by inactivating the RNA-dependent RNA polymerase, quinic acid derivatives inhibited the replication of DENV-3 assayed using a sub-genomic replicon in RepDV3-Huh 7.5 cells^39^. Additionally, anthranilic acid derivatives were reported to act as allosteric inhibitors by binding to HCV NS5B polymerase and inhibiting viral genome replication^40,41^.

Antiviral mechanisms of action already described for some compounds are associated with the ability to interact with viral proteins^42–44^. In the absence of functional ZIKV NS5 RNA polymerase and informed by the observation that viral RNA polymerases present structural and functional similarities^45^, we tested whether FAM E3 could inhibit the synthesis of ZIKV genomic RNA *in vitro* by purified bacteriophage SP6 RNA polymerase. However, no significant inhibition of viral RNA transcription was observed. This indirect data, together with molecular docking results, suggests that the observed replication inhibition was unlikely to be due to the direct inhibition of RNA polymerase activity. Similarly, our results demonstrated that FAM E3 did not intercalate with dsRNA, a mechanism of action described for compounds which inhibit HCV replication, another member of *Flaviviridae* family^45,46^.

Knowing that host cell lipids provide a replication platform for viruses, including members of genus *Flavivirus*, several studies have shown that the antiviral potential of some compounds is related to their interference to the cellular lipid metabolism, and as a consequence prevent viral morphogenesis. We investigated whether FAM E3 interferes with host cell lipid metabolism which could contribute to inhibition of viral replication. However, the results showed that FAM E3 did not interfere in the morphogenesis of lipid droplets (LDs) in non-infected Vero cells. In contrast, treatment of ZIKV infected Vero cells with FAM E3 resulted in a reduction in the numbers of LDs, although this is likely to be a consequence of its anti-ZIKV activity, decreasing replication levels. Heaton *et al*. showed that the NS3 protein of DENV is responsible for recruitment of fatty acid synthase (FASN) to virus replication sites^47^. Since our results suggested that FAM E3 binds to and stabilizes ZIKV NS3^Hel^, we speculate that this interaction may result in a reduction of lipid recruitment to the virus replicase complex, which interferes in viral replication. NS3^Hel^ is a promising target for antiviral drug discovery due to its essential role in the replication of the viral genome^48^. Some examples of helicase inhibitors include ivermectin^49,50^, suramin^51^ and benzoxazole^52^, which presented activity against both YFV^49^ and DENV helicases^51–53^. The *Flavivirus* helicases possess two enzymatic activities: adenosine triphosphatase (ATPase) which provides the chemical energy, and RNA triphosphatase (RTPase) that unwinds viral RNA during the replication process^37^.

In summary, we have shown that the synthetic compound FAM E3 can inhibit ZIKV infection by blocking the genome replication stage. Through molecular docking it was possible to predict a possible interaction between FAM E3 and the ZIKV NS3 helicase, an essential protein for ZIKV replication. Based on this, the thermal stability and the ATPase activity of the helicase domain of ZIKV (NS3^Hel^) were investigated *in vitro* and demonstrated that FAM E3 could bind to and stabilize NS3^Hel^. The results may be useful for further development of antiviral against ZIKV infection, as well as for a better understanding of how exactly this synthetic compound inhibits viral replication.

## Material and Methods

### Synthesis, purification and structural elucidation of FAM E3

To a mixture of 2-bromo-5-methoxybenzoic acid (20.0 mmol) and 4-methoxyaniline (20.0 mmol) in *n-*pentanol (50 mL) were added 50 mg of copper and 3.0 g of K_2_CO_3_. The mixture was stirred under reflux for 24 hours and monitored by TLC. After cooling, solvent was evaporated under reduced pressure. The powder was solubilized in ethyl acetate (250 mL) followed by liquid-liquid extraction with HCl solution 1,0 mol/L (200 mL × 2). Organic phase was washed with deionized water (200 mL), dried with Na_2_SO_4_ and evaporated under reduced pressure. The crude product was purified by column chromatography over silica gel, using a mixture of ethyl acetate and hexane as mobile phase^45,54^. Structure of FAME3 was confirmed by ^1^H and ^13^C NMR data analyses. NMR spectra were recorded on Varian INOVA-500^®^ (11.7 T) spectrometers, operating at 500 MHz for ^1^H NMR and 126 MHz for ^13^C NMR. Chemical shifts (*δ*) were referenced to non-deuterated solvent signals. Signal multiplicities were reported as singlet (s), broad singlet (brs), doublet (d) and double doublet (dd). Amorphous yellow solid; yield 45%; ^1^H NMR (500 MHz; DMSO-d_6_): *δ*_H_* = *3.70 (s, 5-OCH_3_), 3.74 (s, 4′-OCH_3_), 6.92 (d, J = 2.0 and 9.0, H-3′ and H-5′), 6.97 (d, *J = *9.5, H-3), 7.02 (dd, *J = *3.0 and 9.5 Hz, H-4), 7.12 (d, *J = *9.0 Hz, H-2′ and H-6′), 7.37 (d, *J = *3.0 Hz, H-6), 9.10 (brs, NH). ^13^C NMR (126 MHz; DMSO-d_6_): *δ*_C_ = 55.2 (4′-OCH_3_), 55.4 5-OCH_3_), 112.1 (C-3), 114.3 (C-1), 114.7 (C-3′ and C-5′), 115.2 (C-6), 122.1 (C-4), 123.7 (C-2′ and C-6′), 134.1 (C-1′), 142.8 (C-2), 150.2 (C-5), 155.4 (C-4′), 169.5 (COOH)^45,54^.

The compounds were dissolved in dimethyl sulfoxide (DMSO) and stored at −20 °C. Dilutions of the compounds in complete medium were made immediately prior to the experiments to reach a maximum final concentration of 0.1% DMSO. For all the assays performed, control cells were treated with medium added of DMSO at the final concentration of 0.1%. Obatoclax (OLX) at 0.125 µM (AdooQ Bioscience) was used as a positive control for inhibition of ZIKV infectivity^55^.

### ZIKV construction

The ZIKV-Nanoluciferase (Nanoluc) construct **(**Fig. 1A**)** used in these assays was described previously^34^. For maintenance and propagation of the plasmid containing the pCCI-SP6-ZIKV-Nanoluc, the *E. coli* Turbo strain (New England Biolabs) was used.

Complete amplification of the viral genome was performed using a PCR reaction with Phusion High Fidelity (Thermo Fisher) enzyme and the designed primers ZIKV-Forward (5′ CG ATT AAG TTG GGT AAC GCC AGG GT 3′) and ZIKV-Reverse (5′ T AGA CCC ATG GAT TTC CCC ACA CC 3′). The PCR product containing SP6 promoter followed by complete viral cDNA was purified with the DNA clean and concentration kit (Zymo Research). *In vitro* transcription was performed using the RiboMAX™ Large-scale RNA Production Systems kit (Promega), as instructed by the manufacturers.

## Cell culture

Vero cells were cultured in Dulbecco’s modified Eagle’s medium (DMEM; Sigma–Aldrich) supplemented with 100 U/mL penicillin (Gibco Life Technologies), 100 mg/mL streptomycin (Gibco Life Technologies), 1% (v/v) non-essential amino acids (Gibco Life Technologies) and 10% (v/v) fetal bovine serum (FBS; Hyclone) at 37 °C in a humidified 5% CO_2_ incubator.

### Cell viability assay

Cell viability was measured by MTT [3-(4, 5-dimethylthiazol-2-yl)-2, 5-diphenyl tetrazolium bromide] (Sigma–Aldrich) method. Vero cells were seeded in a 96-well plate at a density of 1 × 10^4^ cells per well and incubated overnight at 37 °C in a humidified 5% CO_2_ incubator. Drug-containing medium at different concentrations was added to the cell culture. After 72 h of incubation at 37 °C, the media was removed and a solution containing MTT at the final concentration of 1 mg/mL was added to each well, incubated for 30 min at 37 °C in a humidified 5% CO_2_ incubator after which media was replaced with 100 μL of DMSO to solubilize the formazan crystals. Absorbance was measured by optical density (OD) of each well at 562 nm, using a spectrophotometer. Cell viability was calculated according to the equation (T/C) × 100%, where T and C represent the mean optical density of the treated group and vehicle control group, respectively. The cytotoxic concentration of 50% (CC_50_) was calculated using Prism (Graph Pad).

### Virus assays

For virus rescue, 8 × 10^6^ Vero cells were electroporated with 10 µg of RNA viral transcript using 4 mm cuvettes (450 V, 600 µF, two pulses with an interval of 8 seconds). After electroporation, cells were suspended in culture media supplemented with 2% FBS and placed into 25 cm^2^ cell culture flask and monitored for signs of infection during 5 days. At the end of this time, the viral supernatant was collected and stored at −80 °C. To determine the viral titer, Vero cells at a density of 3 × 10^5^ per well were seeded in a 6 well plate for 24 h prior to infection. Cells were infected with ZIKV-Nanoluc at 10-fold serially dilutions for 2 h at 37 °C. The inoculum was removed and the cells were washed with PBS to completely remove the unbound virus followed by addition of cell culture media supplemented with 2% FBS and 2% carboxymethyl cellulose (CMC). Infected cells were incubated for 5 days at a CO_2_ incubator at 37 °C. The media was removed and cells were fixed with 4% formaldehyde, stained with 0.5% violet crystal and the plaques were counted.

### Antiviral assays

For the initial screening of compounds, Vero cells were seeded at density of 1 × 10^4^ cells per well into 96-well plates 24 h prior to the infection. ZIKV-Nanoluc at a multiplicity of infection (MOI) of 0.1 and compounds were simultaneously added to cells. Samples were harvested in *Renilla* luciferase lysis buffer (Promega) at 72 h post-infection (h.p.i.) and virus replication was quantified by measuring Nanoluciferase activity using the *Renilla* luciferase Assay System (Promega) (Fig. 1B). The effective concentration of 50% inhibition (EC_50_) was calculated using Prism (Graph Pad). The values of CC_50_ and EC_50_ were used to calculate the selectivity index (SI = CC_50_/EC_50_). 0.1% DMSO and 0.125 µM OLX were used as vehicle and positive controls, respectively.

To evaluate the dose-dependence of the antiviral effect, FAM E3 at concentrations ranging from 1 µM to 10 µM, and ZIKV-Nanoluc (MOI = 0.1) were added to the cells simultaneously for 72 h. The cells were washed with PBS and harvested in *Renilla* luciferase lysis buffer prior to measurement of luminescence. Cell viability was analyzed concomitantly.

The effect of FAM E3 against a wild type ZIKV strain was tested by using the primary clinical isolate of ZIKV (provided by the Evandro Chagas Institute in Belém, Pará^35^) (ZIKV^BR^). Vero cells were infected with ZIKV^BR^ at MOI = 0.1 and treated with FAM E3 3 µM or controls for 72 h. Then, intracellular virus was titrated by analysing focus-forming units per milliliters (Ffu/mL).

### Time-of-addition assay

To assess the effect of FAM E3 on ZIKV entry to the host cells, inoculum containing ZIKV-Nanoluc (MOI = 0.5) and compounds were simultaneously added to cells (1 × 10^4^) and incubated for 2 h at 37 °C. Cells were extensively washed with PBS to completely remove virus and compounds and were replaced with fresh media. Samples were harvested in *Renilla* luciferase lysis buffer (Promega) at 72 h.p.i. and virus infectivity was quantified by measuring luciferase activity using the *Renilla* luciferase Assay System (Promega).

Alternatively, cells were infected with ZIKV-Nanoluc (MOI = 0.5) for 2 h, the viral inoculum was completely removed by extensive washing with PBS, and compounds were added. The inhibition of ZIKV replication was measured by quantifying Renilla luciferase activity 72 h.p.i, as previously described.

Finally, Vero cells at a density of 1 × 10^4^ cell per well were incubated with the compound for 2 h at 37 °C in a humidified 5% prior to infection. After incubation, cells were washed extensively and infected with ZIKV-Nanoluc (MOI = 0.5) for 2 h. Then, the inoculum was removed, cells were washed to completely remove non-endocytosed virus and fresh media was added. At 72 h.p.i cells were analyzed as described above. In all Time-of-Addition experiments DMSO and OLX were used as controls as described above.

### DsRNA intercalation assay

To investigate whether the compound interacts with the dsRNA, an experiment using the previously described protocol was performed^56^. Briefly, fifteen nanograms of the dsRNA were incubated with 3 µM of FAM E3 at room temperature for 45 min and electrophoresed on a 1% agarose gel prior to analysis by densitometry. DMSO and Doxorubicin (DOX) at 100 µM were used as negative and positive control, respectively.

### SP6 RNA polymerase transcription assay

Assuming that viral RNA polymerases have similar topology and functions^45,57^, we used SP6 RiboMAX ™ Large-scale RNA Production Systems kit (Promega) to evaluate the effect of the compound at *in vitro* RNA transcription. The pCCI-SP6-ZIKV-Nanoluc was used as target with the addition of 3 µM FAM E3. Complete amplification and purification of transcripts corresponding to the viral genome was performed as per manufacturers instructions. The RNA was quantified, samples were resolved in a 1% agarose gel, and results were analyzed by densitometry. DMSO was used as control.

### NS3^Hel^ cloning, overexpression and purification

The coding region of NS3^Hel^ from the MR766 strain was cloned into the expression vector pETTrx-1a/LIC by Cellco Biotec, generating the NS3^Hel^_ pETTrx-1a/LIC expression vector. Rosetta (DE3) *E. coli* (Novagen) cells were transformed with NS3^Hel^_ pETTrx-1a/LIC and grown in ZYM 5052 autoinduction medium, supplemented with 50 µg.ml^-1^ kanamycin and 34 µg.ml^-1^ chloramphenicol at 37 °C, until the OD_600_ reached 0.6. Protein was expressed at 18 °C for 24 h. Cells were harvested by centrifugation and cell pellets was resuspended in 20 mM Tris pH 7.0, 500 mM NaCl, 20 mM Imidazole, 10% glycerol. Cells were lysed by sonication and cell debris was separated by centrifugation. NS3^Hel^ was purified using five steps: a HisTrap HP 5.0 mL with a Ni Sepharose resin (GE Healthcare), a buffer exchanged with Histrap Desalting 5 ml (GE Healthcare), a TEV protease cleavage from 6His-TRX-tag, another HisTrap HP 5.0 ml with a Ni Sepharose resin (GE Healthcare) and size-exclusion chromatography on a XK 26/1000 Superdex 200 column (GE Healthcare) pre-equilibrated in buffer 20 mM Bis-Tris pH 7.0, 500 mM NaCl, 10% glycerol. The final protein sample was analyzed in SDS-PAGE 10% to confirm its purity. Concentration was determined spectrophotometrically in a Nanodrop 1000 spectrophotometer (Thermo Scientific).

### Thermal stability assay by differential scanning fluorimetry (DSF)

To investigate the thermal stability of helicase domain of ZIKV (NS3^Hel^), FAM E3 was diluted to 1.25 mM in 100% DMSO (Synth) and Helicase in 20 mM Bis-Tris (Sigma), pH7, 500 mM NaCl (Sigma), 10% glycerol. A solution consisting 20 µM Helicase, 5x Sypro® Orange (Sigma Aldrich) was prepared and transferred into each well of the 96-well assay plate (Axygen). 200 μM of FAM E3 was added and an optical adhesive (Hampton) was used to seal the plate. The thermal stability measurements were performed by monitoring the fluorescence of Sypro® Orange (λ_excitation_ = 490 nm and λ_emission_ = 575 nm) while the samples were heated from 25 to 74 °C at a rate of 1 °C/min in a conventional quantitative PCR instrument Mx3005P. Thermal denaturation curves were obtained using GraphPad Prism 5.0 and an approximation through the Boltzmann equation as described by Huynh and Partch^58^. DMSO was used as reference. The experiments were performed in duplicate.

### NS3^Hel^ NTPase activity assay by malachite green assay

Assays to evaluate the ATPase activity of the NS3^Hel^ were performed using the commercial QuantiChrom™ ATPase/GTPase Assay Kit (BioAssay Systems) as described by Cao *et al*.^59^. The assay was standardized for NS3^Hel^ as described in the kit manual. Protein was incubated in 20 mM Bis-Tris buffer, pH7, 500 mM NaCl, 10% glycerol previously supplemented with 8.0 mM MgCl_2_ (Sigma) in a 96-well plate (Greiner Crystal Clear). FAM E3 at 320 μM in 20 mM Bis-Tris buffer, pH7, 500 mM NaCl, 10% glycerol was added into each well to a final concentration of 40 μM in the reaction. The reaction was started with ATP (Sigma) at 2.0 mM and incubated for 30 min at 25 °C. Reactions were terminated with the addition of reagent buffer supplied by the manufacturer and incubated again for 45 min at room temperature before absorbance measurement at λ = 620 nm, which is associated with amount of phosphate released due to ATP hydrolysis. The tests were performed in duplicates. DMSO (1% vol/vol) was used as reference. The results were analyzed and plotted using the GraphPad Prism 5.0 program.

### Microscale thermophoresis

Experiments were performed on a Monolith® NT.115 (Nanotemper technologies). NS3^Hel^ was labelled on cysteine residues with NT-647-Maleimide dye (Nanotemper Technologies) using the Monolith NTTM Protein Labeling Kit RED-MALEIMIDE as per manufacturer’s instructions. The concentration of protein indicated for MicroScale Thermophoresis experiments was 40 nM and a serial dilution of FAM E3 from 5 mM to 150 nM^60^. The dissociation constant K_d_ was obtained by fitting the binding curve with the Hill function.

### Immunofluorescence assay

For immunofluorescence assay, 2 × 10^5^ Vero cells were grown in 6-well plates 24 h prior infection. ZIKV-Nanoluc (MOI = 0.1) and compounds were simultaneously added to cells. Naïve Vero cells treated with DMSO were used as non-infected control. Cells were fixed at 72 h.p.i with 4% paraformaldehyde and washed with PBS and blocking buffer (BB) containing: 0.1% Triton X-100 (Vetec Labs, BR), 0.2% bovine albumin (BSA) and PBS for 30 min. Then, cells were incubated with primary rabbit polyclonal anti-NS3 antibody diluted in BB for 1 h. Alexa Fluor 594 conjugated anti-rabbit IgG was used as secondary antibody. Cells were washed and labelled for nuclei and lipid droplets (LDs) with DAPI and BODYPI 493/503, respectively. Images were analyzed at EVOs cell imaging systems fluorescence microscopy (Thermo Fisher Scientific).

### Molecular docking

FAM E3 was docked into six available crystallographic ZIKV protein structures using the Autodock Vina 1.1.2 software^61^. The crystallographic structures of NS2B-NS3 protease (PDB ID: 5H4I)^62^; NS3 helicase (PDB ID: 5GJB^37^, helicase with RNA strand); NS5 methyltransferase (PDB ID: 5KQR)^63^; NS5 polymerase (PDB ID: 5TFR)^64^, capsid protein (PDB ID: 5YGH)^65^ and envelope protein (PDB ID: 5LBV)^66^ were obtained from the Protein Data Bank (https://www.rcsb.org). The target proteins were prepared as part of the OpenZika project^67^ by using the MolProbity^68,69^ server to add the hydrogen atoms; the open source version of PyMOL^70^ was then used to align all target models onto a single coordinate reference frame (using the align by alpha carbons command line); followed by using AutoDockTools 1.5.6^71^, to format the atom types, calculate Gasteiger-Marsili charges, and merge the non-polar hydrogens onto their respective heavy atoms, using the default AutoDockTools preparation protocol for proteins, described elsewhere^71^.

The SMILES structure of FAM E3 was obtained from the PubChem database (https://pubchem.ncbi.nlm.nih.gov/). Then, the ligand was prepared in the Avogadro software 1.2.0^72^, by adding hydrogen atoms at pH 7.4 and minimizing the geometry using the MMFF94 force field. The minimized structure was then prepared in AutoDockTools 1.5.6^71^, following the standard preparation protocol for ligands (allowing full ligand flexibility)^71^.

The protein grid coordinates were built based on ZIKV protein binding pockets described in the literature: NS2B-NS3 protease (pocket of co-crystallized inhibitor boronate^73^ and ((1H-benzo[*d*]imidazole-1-yl) methanol))^62^, NS3 helicase (RNA and ATP binding sites), NS5 methyltransferase (Guanosine-5′-triphosphate (GTP), S-Adenosyl methionine (SAM) and active), NS5 polymerase (RNA, nucleoside triphosphate (NTP) and active site) and E protein (predicted pocket)^74^. The capsid pockets were predicted using the PockDrug server^75^: pocket 1 (between *N*-terminal - α1 helix of the monomers) and pocket 2 (between α4 helices of the monomers)^48^. The analysis of docking results was based on docking scores, 2D protein-ligand interaction map and visual inspection of the docked 3D binding modes. Visual Molecular Dynamics program (VMD)^76^ was used to render the 3D molecular image.

### Statistical analysis

Individual experiments were performed in triplicate and all assays were performed a minimum of three times in order to confirm the reproducibility of the results. Differences between means of readings were compared using analysis of variance (one-way or two-way ANOVA) or Student’s *t*-test using Graph Pad Prism 5.0 software (Graph Pad Software). *P* values of less than 0.05 (***) were considered to be statistically significant.

## Supplementary information

Supplementary Table S1. Diarylamines derived from anthranilic acid (FAMs).
