# Supplementary material for: A diarylamine derived from anthranilic acid inhibits ZIKV replication
Source: Sci Rep. 2019 Nov 27;9:17703. doi: 10.1038/s41598-019-54169-z (PMC6881388; doi:10.1038/s41598-019-54169-z)
Supplement: Supplementary file 1 — Supplementary Table S1. Diarylamines derived from anthranilic acid (FAMs). [file 41598_2019_54169_MOESM1_ESM.docx]

**A diarylamine derived from anthranilic acid inhibits ZIKV replication**

**Suely Silva^a,b^,** Jacqueline Farinha Shimizu^a,b^; Débora Moraes de Oliveira^a^, Leticia Ribeiro de Assis^c^; Cintia Bittar^b^; Melina Mottin^d^; Bruna Katiele de Paula Sousa^d^; Nathalya Cristina de Moraes Roso Mesquita^e^; Luis Octávio Regasini^c^; Paula Rahal^b^;, Glaucius Oliva^e^; Alexander Luke Perryman^f^, Sean Ekins^g^; Carolina Horta Andrade^d^; Luiz Ricardo Goulart^h^; Robinson Sabino-Silva^i^; Andres Merits^j^; Mark Harris^k^; Ana Carolina Gomes Jardim ^a,b*^

**SUPPLEMENTARY MATERIAL**

**Supplementary Table S1. Diarylamines derived from anthranilic acid (FAMs).**

| Compound | Concentration µM | Cell viability (%) | Infectivity (%) |
| --- | --- | --- | --- |
| **FAM-A3** | **10** | **107** | **99** |
| **FAM-A3'** | **2** | **119** | **119** |
| **FAM-A5** | **10** | **115** | **110** |
| **FAM B3** | **10** | **108** | **56** |
| **FAM B4** | **40** | **77** | **49** |
| **FAM C1** | **50** | **100** | **61** |
| **FAM C3** | **10** | **119** | **94** |
| **FAM C5** | **10** | **113** | **98** |
| **FAM D1** | **30** | **62** | **20** |
| **FAM E1** | **10** | **118** | **99** |
| **FAM L3** | **10** | **112** | **101** |
| **FAM I5** | **50** | **62** | **40** |
| **FAM G3** | **0,4** | **114** | **118** |
| **FAM F3** | **10** | **114** | **117** |
| **FAM E3** | **10** | **61** | **1** |
| **FAM F5** | **50** | **114** | **108** |
| **FAM D5** | **50** | **102** | **94** |
| **FAM A1** | **10** | **111** | **94** |
| **FAM B1** | **50** | **101** | **122** |
| **FAM D3** | **2** | **140** | **131** |
| **FAM E5** | **50** | **101** | **66** |
